# MiR-34a-5p Inhibits Proliferation, Migration, Invasion and Epithelial-mesenchymal Transition in Esophageal Squamous Cell Carcinoma by Targeting LEF1 and Inactivation of the Hippo-YAP1/TAZ Signaling Pathway

**DOI:** 10.7150/jca.39861

**Published:** 2020-03-04

**Authors:** Xinyu Wang, Yue Zhao, Qijue Lu, Xiang Fei, Chaojing Lu, Chunguang Li, Hezhong Chen

**Affiliations:** Department of Thoracic Surgery, Changhai Hospital, Second Military Medical University, Shanghai 200433, China

**Keywords:** miR-34a-5p, lymphoid enhancer-binding factor 1 (LEF1), esophageal squamous cell carcinoma (ESCC), epithelial-mesenchymal transition (EMT)

## Abstract

**Background**: Our previous studies reported that lymphoid enhancer-binding factor 1 (LEF1) was upregulated in esophageal squamous cell carcinoma (ESCC) and the positive expression of LEF1 was correlated with aberrant clinicopathological characteristics in ESCC patients. However, the upstream mechanism of regulating LEF1 is not clear fully. In this study, we explored the role of miR-34a-5p in ESCC and the possible regulatory mechanism.

**Methods**: In this study, we applied western blotting, quantitative real-time polymerase chain reaction (qRT-PCR), bioinformatics analysis, a luciferase reporter assay, and a series of functional assays to show the potential role of miR-34a-5p in regulating LEF1 in ESCC.

**Results**: By various functional assays, we demonstrated that LEF1 promoted proliferation, migration, invasion and epithelial-mesenchymal transition (EMT) in ESCC cells. By bioinformatics analysis and luciferase reporter assay, miR-34a-5p was identified for directly targeting LEF1. Then we investigated the expression of miR-34a-5p and LEF1 in ESCC. As a result, miR-34a-5p was downregulated while LEF1 was upregulated in ESCC tissue and cell lines. Overexpression of miR-34a-5p could inhibit proliferation, migration, invasion and EMT of ESCC cells. The rescue experiment showed that re-expression of LEF1 reversed the suppressive effect caused by miR-34a-5p. At last, we found that miR-34a-5p could suppress Hippo-YAP1/TAZ signaling pathway in ESCC.

**Conclusion**: Our results indicate miR-34a-5p inhibits proliferation, migration, invasion and EMT in ESCC by targeting LEF1 and suppressing the Hippo-YAP1/TAZ signaling pathway, which may provide a new antitumor strategy to delay ESCC progress.

## Introduction

Esophageal cancer (EC) is the 6th leading cause of death from cancer and the 8th most common cancer worldwide 1. The 5-year survival of EC in most countries is lower than 30%, except Japan and South Korea 2. Esophageal adenocarcinoma (EAC) is the main histologic subtype in west countries while esophageal squamous cell carcinoma (ESCC) remains the predominant form in China 3. Despite advances in diagnosis and therapy, the prognosis of patients with ESCC remains poor, is mainly associated with recurrence and metastasis 4. Therefore, it is imperative to explore some novel therapeutic targets to inhibit tumor progression for ESCC patients.

Lymphoid enhancer-binding factor 1 (LEF1) is a key downstream mediator of the activated Wnt/ β-catenin signaling pathway, which regulates tumorigenesis and the progression of multiple tumours, such as breast cancer, lung adenocarcinoma, colon cancer, prostate cancer and leukemia 5-9. Nevertheless, there are limited studies on the role of LEF1 in ESCC. Our previous study reported that LEF1 was predominantly expressed in ESCC cell lines and tumor tissues and that the positive expression of LEF1 was correlated with aberrant clinicopathological characteristics in ESCC patients 10. In addition, we also found LEF1 could promote tumorigenicity by activating the TGF-β signaling pathway in ESCC 11. Such important role of LEF1 in ESCC shall arouse our interests in exploring the further mechanisms of LEF1 regulation. Herein, we aimed to elucidate how LEF1 expression was regulated and explore the upstream mechanism facilitating its effect on the progression in ESCC.

miRNAs are members of small noncoding RNAs consisting of 19 to 24 nucleotides in length. They interact with the 3'-untranslated region (3'-UTR) of target mRNAs and repress protein expression. Given their significant roles in tumour invasion, metastasis and recurrence, identification of key candidate miRNAs could be further investigated as therapeutic targets in cancers 12.

In this study, we identified LEF1 as a direct target of miR-34a-5p via binding to the 3'-UTR of LEF1. Inhibition of LEF1 by miR-34a-5p could suppress the migration, invasion and epithelial-mesenchymal transition (EMT) of ESCC. What's more, miR-34a-5p could inactivate Hippo-YAP/TAZ signaling pathway. Targeting miR-34a-5p and LEF1 may provide a new antitumor strategy to delay ESCC progress.

## Methods

### Clinical tissue

Sixteen fresh patient specimens were collected from patients who were diagnosed with primary ESCC and who received radical esophageal surgery without preoperative chemoradiotherapy from March 2019 to May 2019 at Changhai Hospital (Shanghai, China). This study was approved by the Ethics Committee of Changhai Hospital. All patients provided written informed consent when admission.

### Cell lines and culture conditions

The human Esophageal Epithelial Cells (HEEC), Eca109 and TE1 cells were purchased from the Shanghai Cell Bank (Shanghai, China). All cell lines cultured in Dulbecco's modified Eagle's medium (Gibco, CA, USA) supplemented with 10% heat-inactivated foetal bovine serum (Gibco-BRL) and antibiotics (100 U/ml penicillin and 100 U/ml streptomycin; HyClone Laboratories, Inc., USA).

### Western blotting

Whole cultured cells were homogenized in 0.1% SDS and 1 mM PMSF (phenylmethylsulfonyl fluoride) and centrifuged at 12, 000 g for 10 min. Protein extracts were subjected to SDS-PAGE and analyzed using the following primary antibodies: LEF1 antibody (Abcam, ab137872), YAP1 antibody (Abcam, ab52771), TAZ antibody (Proteintech, 23306-1-AP), E-cadherin antibody(Abcam, ab40772), N-cadherin antibody (Abcam, ab18203) and GADPH antibody (Abcam, ab8245). Then, the membranes were incubated with secondary antibodies (CST,7076,7074) at room temperature for 1 hour. All experiments were performed in triplicate.

### RNA extraction and quantitative RT-PCR (qRT-PCR)

Total RNA was extracted from cultured ESCC cell lines using Trizol (Invitrogen, Grand Island, NY) according to the manufacturer's instruction. The cDNA was synthesized using the PrimeScript RT Reagent Kit (TaKaRa Bio, Shiga, Japan) following the manufacturer's instructions. Real-time PCR was performed on a Roche Light Cycler 480 (Roche) using SYBR Green PCR Master Mix (TaKaRa Bio, Shiga, Japan). Each measurement was performed in triplicate and the results were normalized by the expression of the GADPH gene. Fold change relative to mean value was determined by 2^-△△Ct^. All experiments were performed in triplicate. Primer sequences are listed in Table 1.

### Bioinformatics analysis

We utilised mirwalk database 13 (http://zmf.umm.uni-heidelberg.de/apps/zmf/mirwalk/micrornapredictedtarget.html), which integrated five commonly used miRNA databases (TargetScan, Pictar, miRwalk, miRDB, and miRanda) to scan for the potential miRNAs that may target 3'-UTR of LEF1 mRNA.

### Wound healing assay

Eca109 or TE1 cells were seeded in six-well plates. Three longitudinal scratches were made with sterile 100-uL pipette tips 48 h after transfection. Then, floating cell debris was washed three times with PBS. Subsequently, the cells were cultured in serum-free medium. Typical wound healing images were observed and photographed at 0 h and 24 h under an inverted microscope.

### Cell proliferation assays

Infected cells were seeded in 96-well plates (2000 cells/well) in triplicate and cultured for 3 days to assess proliferation with the Cell Counting Kit-8 (CCK-8, bimake, USA). The absorbance was measured at 450 nm.

### Cell migration and invasion assay

Cell migration and invasion ability was assessed by 24-well transwell chambers (Corning) in the presence or absence of Matrigel (Corning, Bedford, MA, USA) coating. Forty-eight hours after transfection, the cells were trypsinized and counted. Approximately 5*10^4^ cells resuspended in 200-uL serum-free DMEM were seeded into the upper chambers, whereas the bottom chamber was filled with 500-uL 10% FBS medium. Twenty-four hours later, non-migrated/non-invaded cells were wiped off with acotton bud, and migrated/invaded cells underneath the chamber were fixed with 95% ethyl alcohol and stained with 0.1% crystal violet.

### miR, siRNA and expression plasmids

Eca109 and TE1 cells were transfected with miR-34a-5p mimic, miR-34a-5p inhibitor and shRNA targeting LEF1 (sh-LEF1) using Lipofectamine 2000 (Invitrogen; Thermo Fisher Scientific Inc., USA). miRs and their corresponding negative controls were synthesized by GenePharma (Shanghai, China). We constructed lentiviral vectors encoding the human LEF1 gene or green fluorescent protein (GFP) in the pLenti-EF1a-EGFP-P2A-Puro-CMV-MCS-3Flag vector (HeYuan Bio-technology Co., Shanghai, China) and designated them as LV-ov-LEF1 or LV-GFP. Stable ESCC cells knockdown of LEF1 were generated using lentiviral constructs expressing sh-LEF1 (GCTACATATGCAGCTTTAT) and negative control (HeYuan Bio-technology Co., Shanghai, China). For the rescue study, cells were co-transfected with miR-34a-5p mimic and LEF1 expression plasmid without the 3 ׳UTR (to avoid downregulation by miR-34a-5p). Cells were harvested at 48h after transfection for RNA analysis and 72h after transfection for protein analysis as previously described.

### Luciferase reporter assay

The wild-type sequence containing the predicted target sites of miR-34a-5p in the 3 ׳UTR of LEF1 mRNA was synthesized by Heyuan Bio-technology Company (Shanghai, China). The pmirGLO-LEF1-wt or pmirGLOLEF1-mut vector was co-transfected with miR-34a-5p mimic or miR-34a-5p negative control into HEK293T cells. Twenty-four hours after transfection, activities of firefly luciferase and Renilla luciferase were measured according to the manufacturer's instruction of the Dual-Luciferase Reporter Assay system (Promega, Madison, WI, USA). The luciferase reporter assay was independently repeated three times.

### Statistical analysis

Data analysis was carried out using IBM SSPS Statistics Version 24 (IBM Corp., Armonk, NY, USA). Data were reported as mean±SD and t-test was used to determine differences between groups. Correlations were analyzed with Pearson's correlation. Differences were considered statistically significant when P < 0.05.

## Results

### LEF1 promoted proliferation, migration, invasion and EMT of ESCC cells

**Figure 1:**
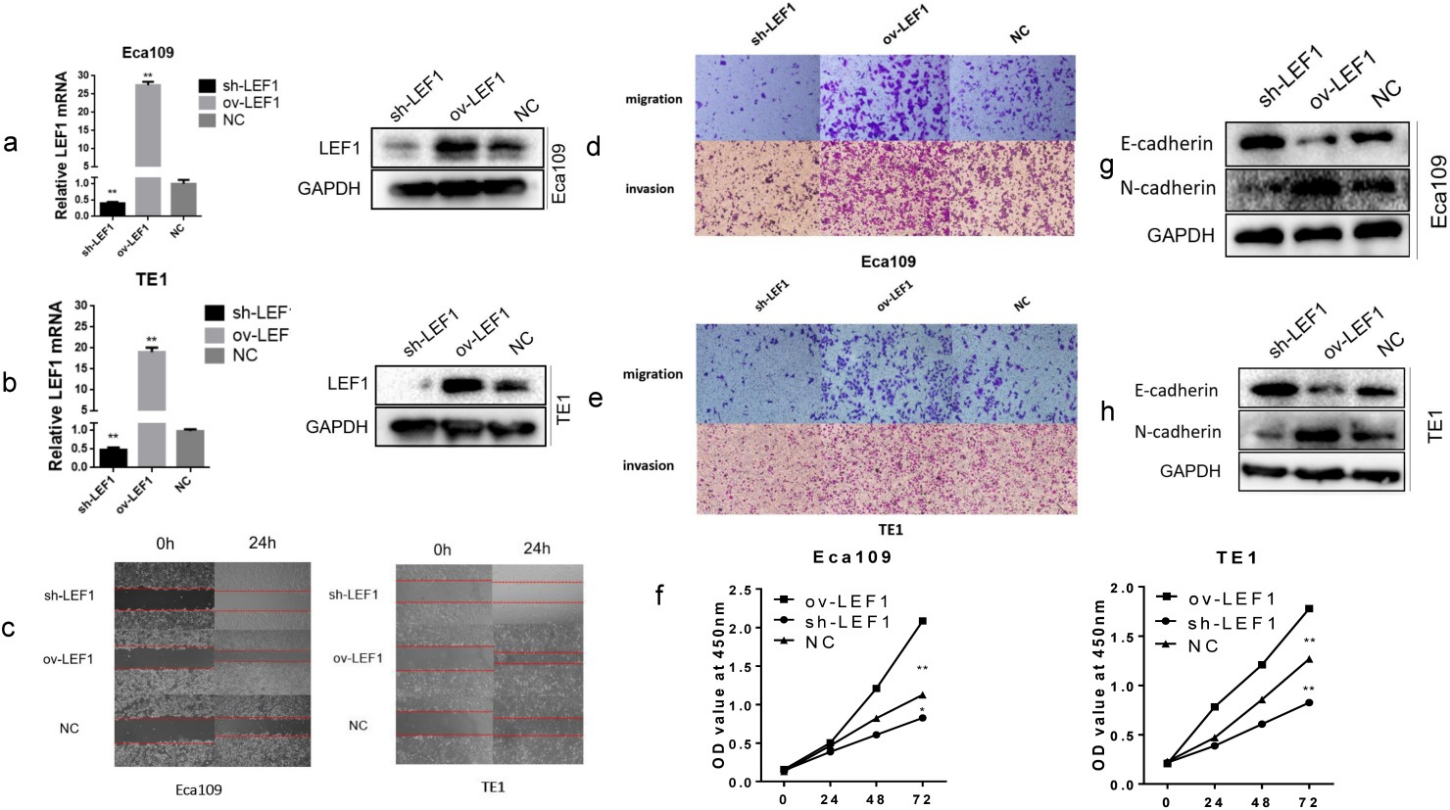
** LEF1 promoted proliferation, migration, invasion and EMT of ESCC cells. (a,b)** The mRNA and protein expression of stably express LEF1 (ov-LEF1) or shLEF1 (sh-LEF1) in ESCC cells was detected by qRT-PCR and western blotting.** (c)** Overexpression of LEF1 increased the mobility while knockdown of LEF1 suppressed the mobility of ESCC cells, as assessed by the wound healing assay (scale bar, 200 μm).** (d,e)** LEF1 overexpression promoted the migratory and invasive abilities while knockdown of LEF1 suppressed the migratory and invasive abilities *in vitro* (scale bar, 100 μm). **(f)** The cell proliferative ability of ESCC cells with LEF1 overexpression (knockdown) was increased (decreased) as indicated by the CCK-8 assay.** (g,h)** LEF1 overexpression decreased levels of E-cadherin and increased levels of N-cadherin was detected by western blotting. *P<0.05, **P<0.01

To investigate the biological effects of LEF1 in ESCC cells, we applied a lentivirus-based approach to stably express LEF1 (ov-LEF1) or shLEF1 (sh-LEF1) in EAC109 and TE1 cells, and the expression level of LEF1 was examined by qRT-PCR and western blotting. Cells infected with LV-GFP were used as negative control (NC). The level of mRNA and protein were increased significantly in the ov-LEF1 cells while those were decreased in the sh-LEF1 cells, as compared with the NC cells (Fig. 1a, b).

To explore the migratory and invasive abilities of LEF1 in ESCC cells, we performed the wound healing and transwell assay. The results revealed that LEF1 overexpression in Eca109 and TE1 cells increased the mobility, migratory and invasive abilities compared to the NC cell group. However, knockdown of LEF1 in Eca109 and TE1 cells decreased the migratory and invasive abilities compared to those in the corresponding groups (Fig. 1 c, d, e and Supplement Fig. S1). The CCK-8 assays indicated that LEF1 overexpression promoted the proliferation of ESCC cells (Fig. 1f).

Next, we evaluated the impact of LEF1 on tumor cell EMT. The results of western blotting revealed decreased levels of E-cadherin and increased levels of N-cadherin in the ov-LEF1 group, while increased levels of E-cadherin and decreased levels of N-cadherin in the sh-LEF1 group, indicating LEF1 could enhance EMT capacity (Fig. 1g, h).

### LEF1 was a direct target of miR-34a-5p in ESCC cells

**Figure 2:**
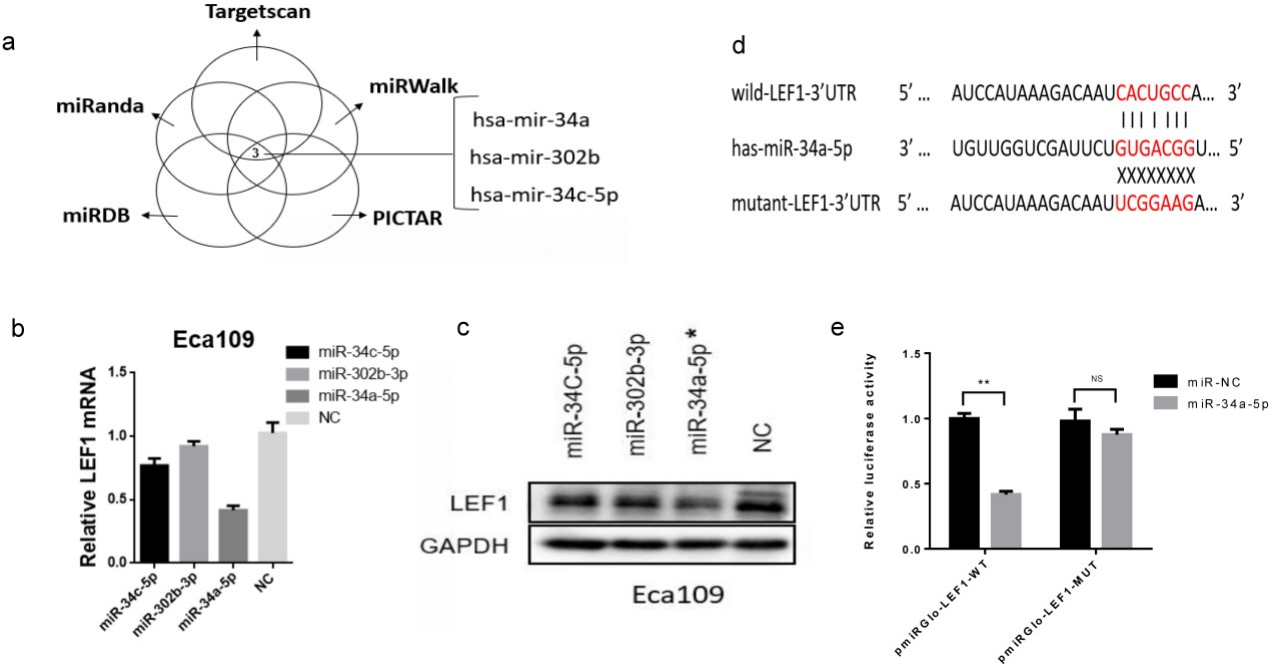
** LEF1 was a direct target of miR-34a-5p in ESCC cells. (a)** miR-34a, miR-302b, miR-34c-5p showed the most likely binding with the 3'-UTR of LEF1 as predicted by mirwalk database. **(b, c)** qRT-PCR and western blot showed LEF1 expression levels in the Eca109 cells transfected with the top three miRNAs mimics (miR-34a-5p, miR-302b-3p, miR-34c-5p) and negative control (NC), indicating miR-34a-5p induced the greatest decline of LEF1 expression. **(d)** Sequences of LEF1 3'-UTR and miR-34a-5p according to the prediction of TargetScan. Wild-type and mutated-type binding sequences of LEF1 3 ׳UTR were shown. **(e)** Luciferase assay on ESCC cells showed that miR-34a-5p markedly suppressed luciferase activity of wild-type reporter constructs. *P<0.05, **P<0.01

To investigate whether miRNAs were involved in regulating LEF1 expression, we identified the potential miRNAs that target 3'-UTR of LEF1 mRNA. As a result, the top three miRNAs (hsa-miR-34a, hsa-miR-302b, hsa-miR-34c-5p) showed the most likely to target LEF1 (six sources, Fig. 2a and Supplement Fig. S2). And meanwhile, Jiao DM et al found miR-34a-5p/miR-34c-5p/miR-302b-3p-LEF1-CCND1/WNT1/MYC axis may be a crucial mechanism in inhibition of cancer metastasis 14. Thus, miR-34a-5p/ miR-302b-3p/ miR-34c-5p were selected out for further study.

We transiently transfected the three miRNAs mimics into Eca109 cell and evaluated LEF1 expression levels using qRT-PCR and western blotting, which showed that miR-34a-5p induced the greatest decline of LEF1 expression (Figure. 2b, c, d).

Next, to validate that LEF1 was a direct target of miR-34a-5p, a dual-luciferase reporter assay was performed. Results showed that co-transfection of pmirGLO-LEF1-wt and miR-34a-5p mimic led to a significant decrease in luciferase activity compared with the NC group, whereas co-transfection of pmirGLO-LEF1-mut and miR-34a-5p mimic had no effect on luciferase activity (Fig. 2e).

**Figure 3:**
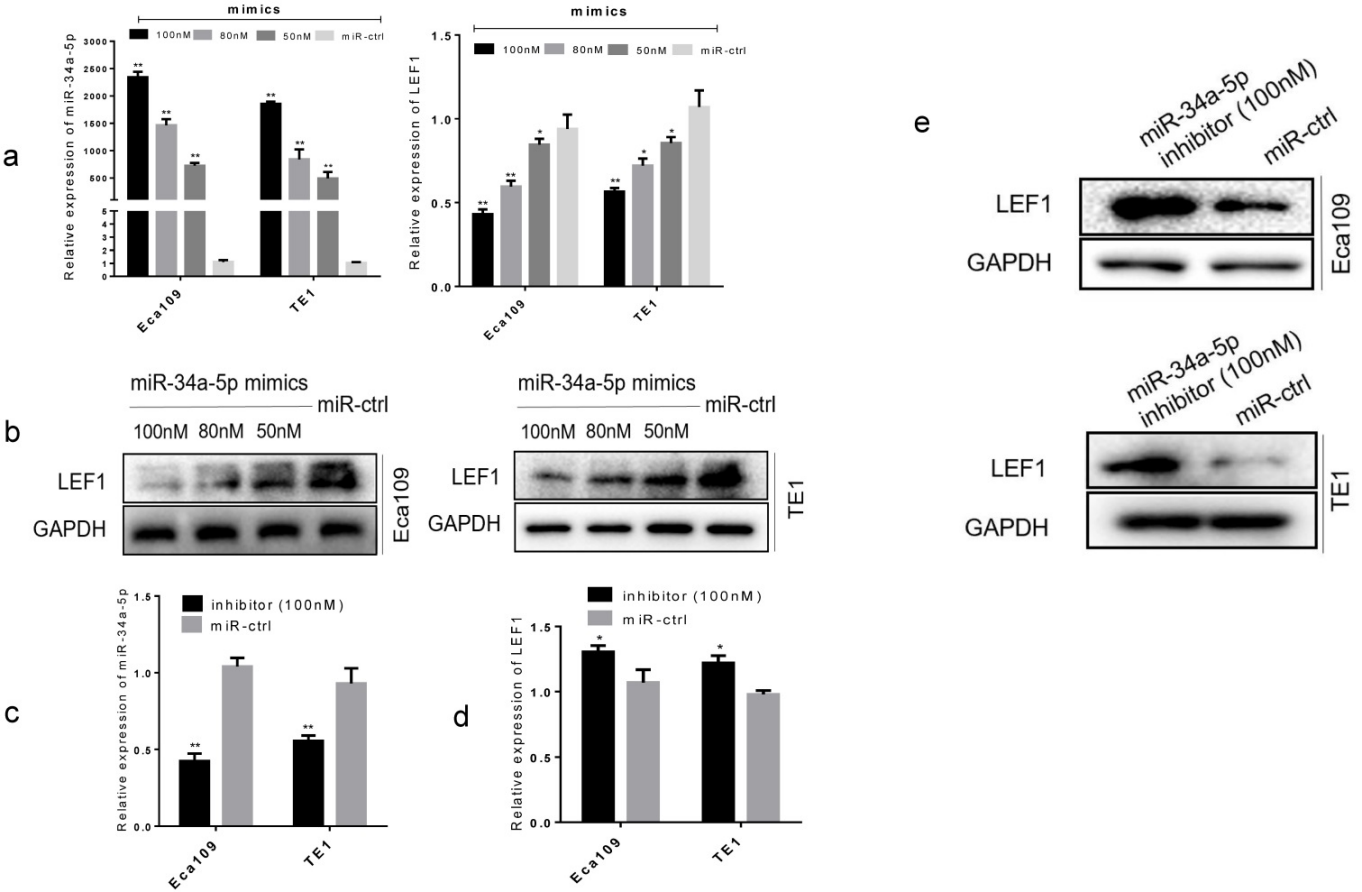
** miR-34a-5p decreased the LEF1 expression levels in a dose-dependent manner. (a)** Left panel showed relative miR-34a-5p expression in Eca109 and TE1 cells after transfection with various concentrations of miR-34a-5p mimic (50, 80, and 100 nM) or miR-ctrl analyzed by qRT-PCR. Right panel showed the corresponding relative LEF1 mRNA expression analyzed by qRT-PCR. **(b)** Western blotting analysis of LEF1 protein levels after cells were transfected with miR-34a-5p mimics in a dose-dependent manner. **(c-e)** Relative miR-34a-5p expression in Eca109 and TE1 cells after transfection with miR-34a-5p inhibitor (100nM) or miR-ctrl analyzed by qRT-PCR and western blotting. *P<0.05, **P<0.01

To further confirm the functional effect of miR-34a-5p on LEF1, we overexpressed miR-34a-5p in Eca109 and TE1 cell lines. Results showed that forced transfection of miR-34a-5p mimic (Fig. 3a), meanwhile it caused a significant decrease in LEF1 expression both at mRNA and protein levels in a dose-dependent manner (Fig. 3a, b). Conversely, an obvious increase in LEF1 expression was observed in Eca109 and TE1 when transfected with miR-34a-5p inhibitor of 100nM (Fig. 3c, d, e).

### miR-34a-5p was downregulated and LEF1 was upregulated in ESCC tissues and cell lines

**Figure 4:**
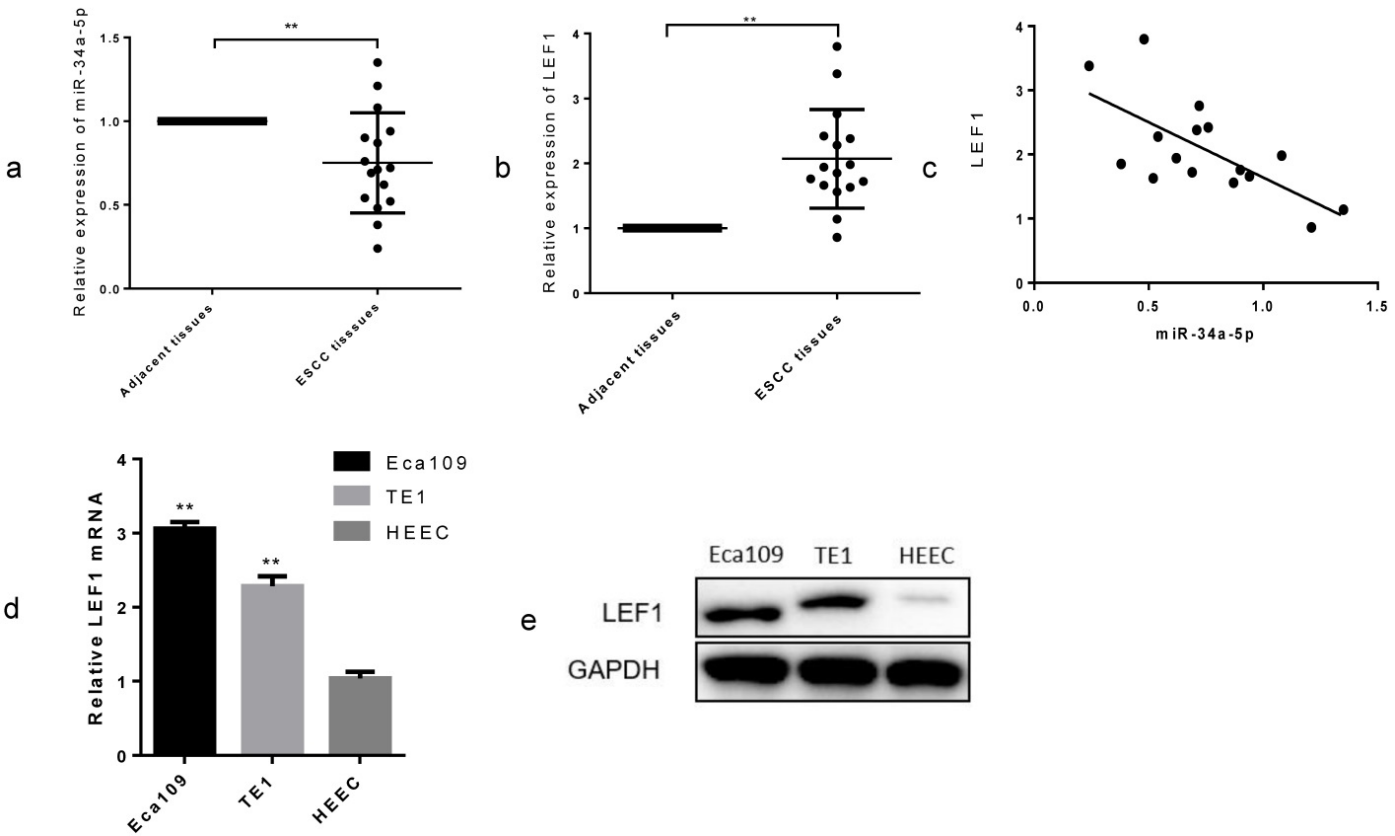
** miR-34a-5p was downregulated and LEF1 was upregulated in ESCC tissues and cell lines. (a,b)** Relative expression of miR-34a-5p and LEF1 in 16 paired ESCC and adjacent normal tissues by qRT-PCR. **(c)** Linear regression analysis between miR-34a-5p and LEF1. **(d,e)** comparison of the LEF1 expression level between ESCC cells and normal human esophageal epithelium cell (HEEC). *P<0.05, **P<0.01

To explore the clinical role in ESCC, we examined the miR-34a-5p expression in 16 paired ESCC tissues and adjacent normal tissues by qRT-PCR methods. As expected, we found that miR-34a-5p was significantly decreased in most of the ESCC tissue compared with that in the matched controls (Fig. 4a, P < 0.01), whereas LEF1 was increased (Fig. 4b, P < 0.01). Linear regression analysis showed a possible relevance between miR-34a-5p and LEF1 in the clinical tissues (Fig. 4c). In addition, we compared the LEF1 expression level between ESCC cells and HEEC using qRT-PCR and WB. LEF1 was obviously upregulated in the Eca109 and TE1 compared with the HEEC (Fig. 4d, e). Clinicopathological analyses showed that a decrease in miR-34a-5p was significantly correlated with advanced T stage, node metastasis and poor differentiation (P = 0.01, P = 0.03, and P < 0.01, respectively, Table 2)**.** No significant association between miR-34a-5p or LEF1 expression level and age or gender was observed. These data indicated that the downregulated miR-34a-5p and the upregulated LEF1 were closely associated with aggressive features of ESCC.

### Overexpression of miR-34a-5p inhibited proliferation, migration, invasion and EMT in ESCC cells

**Figure 5:**
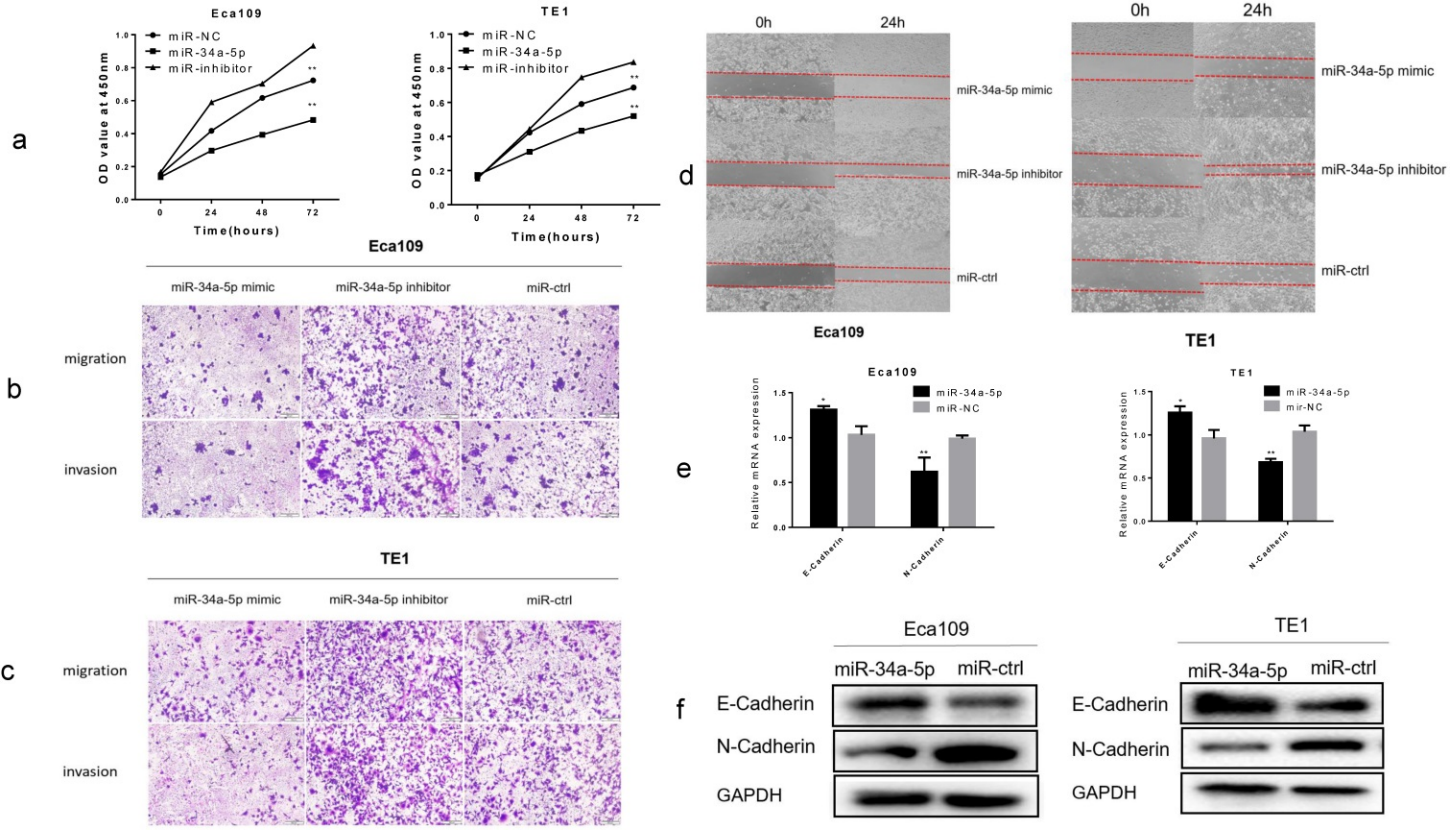
** Overexpression of miR-34a-5p inhibited proliferation, migration, invasion and EMT in ESCC cells. (a)** The cell proliferative ability of ESCC cells treated with miR-34a-5p mimic (miR-34a-5p inhibitor) was decreased (increased) as indicated by the CCK-8 assay. **(b-d)** miR-34a-5p mimic suppressed the mobility, migratory and invasive abilities while miR-34a-5p mimic promoted the mobility, migratory and invasive abilities of ESCC cells. **(e,f)** miR-34a-5p significantly increased the E-cadherin expression and reduced the N-cadherin expression analyzed by qRT-PCR and western blotting. *P<0.05, **P<0.01

Using CCK-8 assay, overexpression of miR-34a-5p significantly inhibited cell proliferation, while downregulation of miR-34a-5p promoted cell proliferation of Eca109 and TE1 cell lines (Fig. 5a). Using Transwell assay, we found that miR-34a-5p overexpression significantly inhibited the migration and invasion of ESCC cells, whereas downregulation of miR-34a-5p promoted the migration and invasion of ESCC cells (Fig. 5b, c and Supplement Fig. S3a, b). Next, we detected cell motility. Results of wound healing assay suggested that miR-34a-5p inhibited motility of Eca109 and TE1 cell lines (Fig. 5d and Supplement Fig. S3c, d). Then we examined the expression level of EMT hallmarks. Our study demonstrated that overexpression of miR-34a-5p significantly increased the epithelial marker of E-cadherin, and reduced the mesenchymal cell marker of N-cadherin (Fig. 5e, f). Taken together, our findings suggested that miR-34a-5p could significantly repress the proliferation, migration, invasion, and EMT of ESCC cells.

### LEF1 rescued the inhibitory effect of miR-34a-5p on ESCC cells

**Figure 6:**
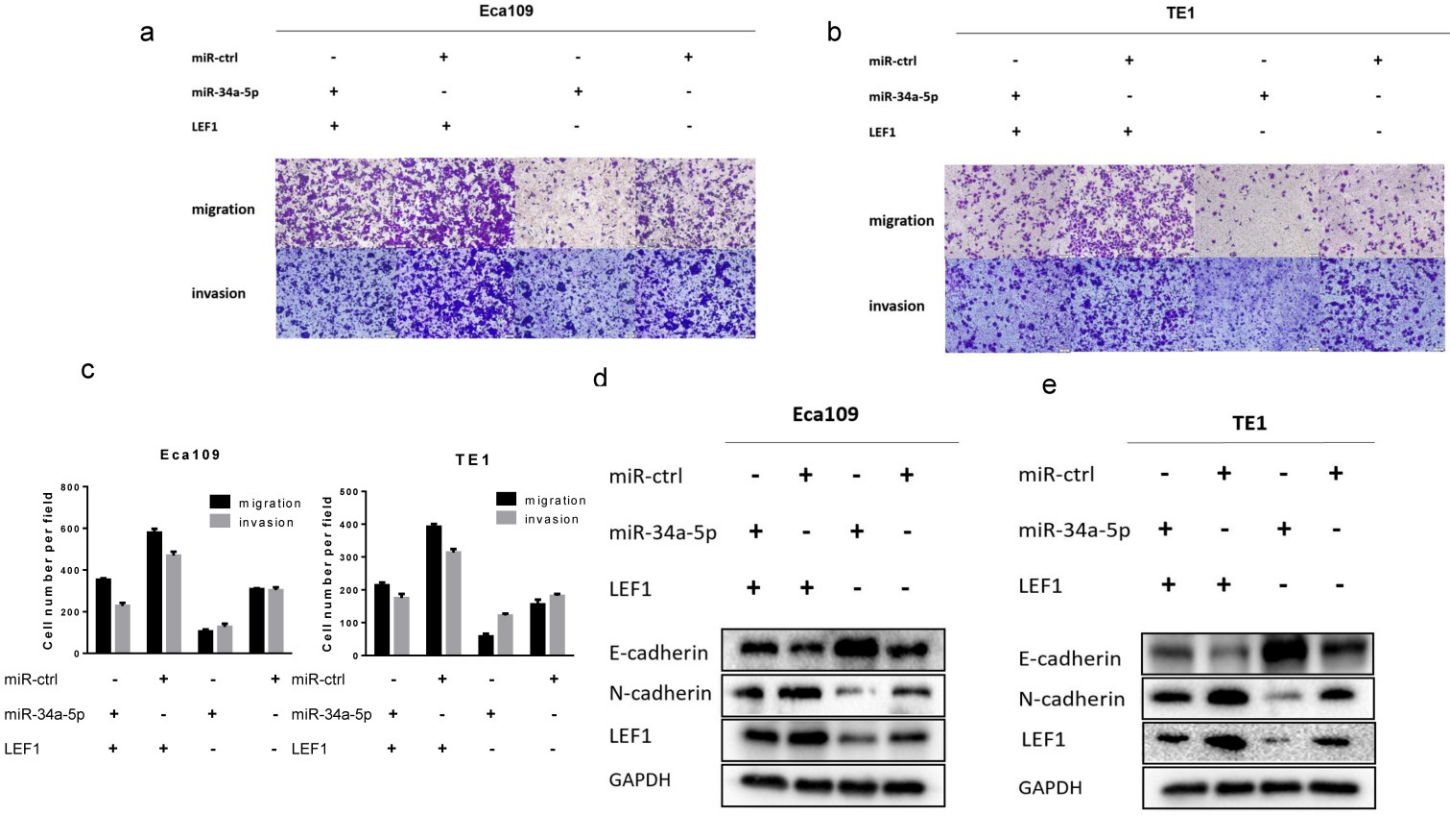
** LEF1 rescued the inhibitory effect of miR-34a-5p on ESCC cells. (a-c)** exogenous LEF1 expression reversed the suppression of migration and invasion caused by miR-34a-4p overexpression (scale bar, 100 μm). **(d, e)** LEF1 reversed the inhibitory effect of miR-34a-5p on EMT markers at the protein levels.

To further demonstrate whether LEF1 could mediate the effect of miR-34a-5p on ESCC cells, a rescue strategy was employed. Four groups of ESCC cells were set: ESCC cells transfected with NC, ESCC cells transfected with miR-34a-5p mimics, ESCC cells transfected with LEF1 expression plasmid, and ESCC cells co-transfected with miR-34a-5p and LEF1-expressing plasmid. Through cell migration and invasion assays, we found that exogenous LEF1 expression reversed the suppression of migration and invasion caused by miR-34a-4p overexpression (Fig 6a, b, c). Consistently, expression of EMT markers was also recovered by overexpression of LEF1. Western blotting analysis showed that the expression of LEF1 inhibited expression of E-Cadherin but restored expression of N-Cadherin (Fig. 6d, e).

### miR-34a-5p could repress Hippo-YAP1/TAZ signaling pathway

**Figure 7:**
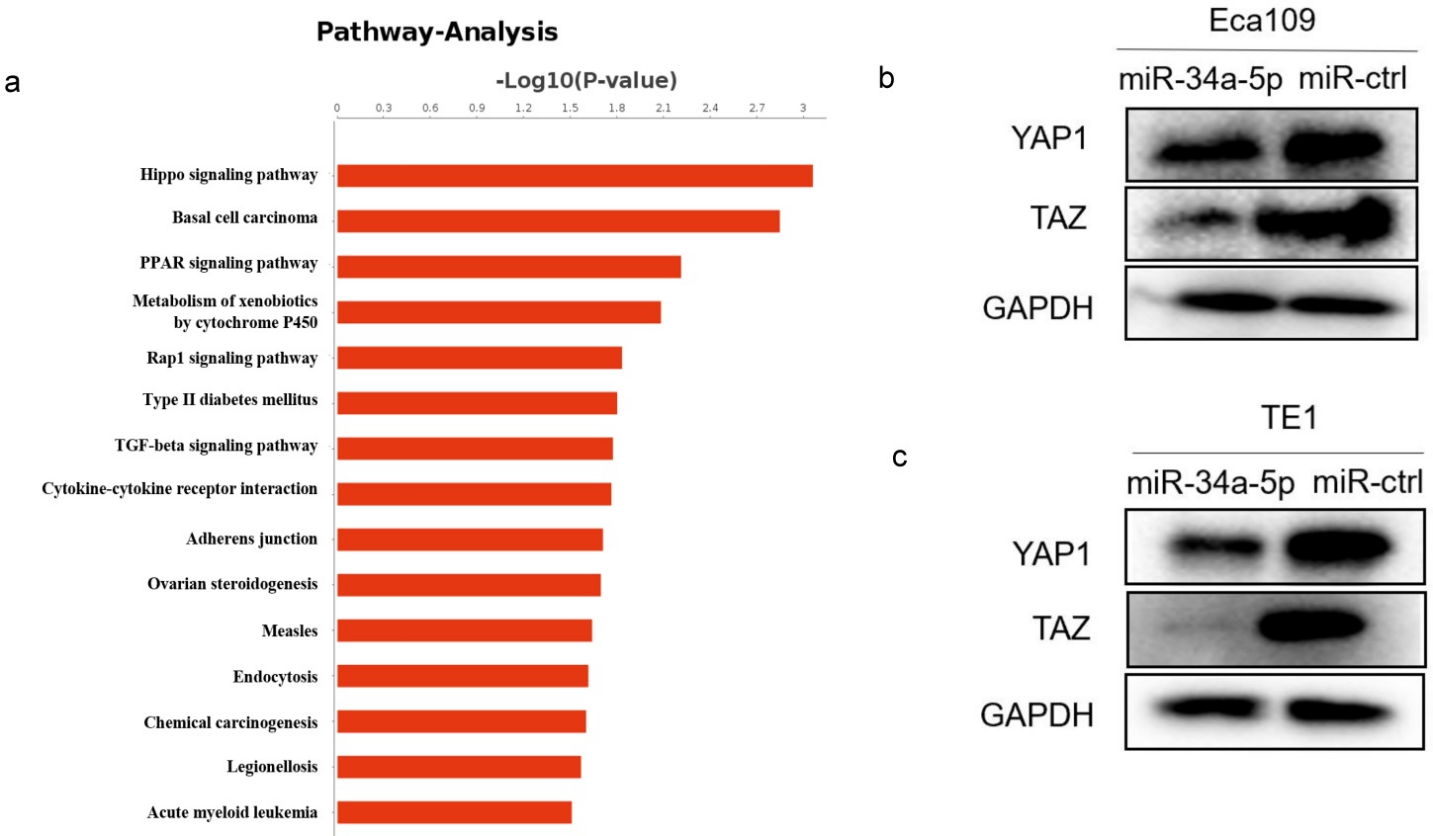
** miR-34a-5p repressed Hippo-YAP1/TAZ signaling pathway. (a)** RNA-Seq data showed that significant change was taken place in Hippo signaling pathway after LEF1 was overexpressed. **(b,c)** YAP1/TAZ expression was downregulated after miR-34a-5p upregulation in Eca109 or TE1 cells compared to the control groups.

Our previous RNA-Seq data showed the most of significantly differentially expressed genes were enriched in the Hippo signaling pathway after LEF1 was overexpressed, indicating LEF1 may regulate Hippo pathway (Fig. 7a). Considering the targeting effect of miR-34a-5p on LEF1, we detected the YAP1/TAZ expression of Hippo signaling pathway by overexpression of miR-34a-5p in ESCC cells. The western blot analysis showed that YAP1/TAZ expression was downregulated after miR-34a-5p upregulation in Eca109 or TE1 cells compared to the control groups (Fig. 7b, c).

## Discussion

EMT involves the generation of mesenchymal-like cells from epithelial cells, which is associated closely with recurrence and metastasis by increasing the viability, proliferative, invasive, and migration abilities in multiple cancers 15,16. LEF1 is essential in stem cell maintenance and organ development, especially in its role in EMT. LEF1 modulates the interaction with EMT hallmarks, such as E-Cadherin, N-Cadherin, Vimentin, and Snail, facilitating the process of EMT 17. In this study, we further reveal that LEF1 could promote the proliferation, migration, invasion and EMT of ESCC, suggesting that the LEF1 could be a therapeutic target for the treatment of ESCC.

MicroRNAs have an important role in biological processes and aberrant miRNA expression is associated with many cancers. For most of miRNAs, they play a role of tumor suppressor, while the rest promote carcinogenesis as oncogenic miRNAs 18. Insights into the roles of miRNAs in cancer have made miRNAs attractive tools and targets for novel therapeutic approaches 19. In our previous studies, we have found that miR-203 and miR-214 inhibited invasion and migration in ESCC, indicating both of them may be potential therapeutic targets for ESCC 20,21.

miR-34a-5p is one of the most characterized tumor suppressor miRNAs in a variety of tumors, which plays a pivotal role in regulating cancer-related processes, such as cell proliferation, apoptosis, EMT, and metastasis 22. In many tumors, miR-34a-5p has been proved downregulated and inhibits tumor progress 23,24. Thus, miR-34a-5p has been considered an ideal therapeutic tool to combat metastasis, chemoresistance and tumor recurrence.

miR-34a-5p is also downregulated in ESCC and the expression level of miR-34a-5p is correlated with clinicopathological feature of the patients 25. In our study, low miR-34a-5p expression was found to be correlated with advanced T stage, node metastasis, and poor differentiation (P = 0.01, P = 0.03, and P < 0.01, respectively). Moreover, *in vitro* trials demonstrated miR-34a-5p inhibited proliferation, migration, invasion, and EMT in ESCC cells. Taken together, we believed that miR-34a-5p could be served as a tumor suppressor in ESCC.

Several target genes regulated by miR-34a-5p were reported to be involved in the process of cell growth, migration and invasion in several malignancies, such as SIRT1, c-Myc, NOTCH1, MMP1, KLF4, BCL2, AGTR1, DLL1, AXL, and CD117 23, 26-29. In the present study, we confirmed that miR-34a-5p could target LEF1 directly by binding to the 3'-UTR of LEF1. By a rescue experiment, we found that ectopic LEF1 expression could reverse the suppression of migration, invasion and EMT caused by miR-34a-4p overexpression, validating the molecular biological function of miR-34a-5p was mediated by suppression of LEF1.

Previous studies have confirmed that miR-34a-5p may regulate Notch signaling pathway 30, PI3K/Akt signaling pathway 31, SIRT1/HIF-1α signaling 32, Wnt/β-Catenin signaling pathway 33. LEF1, a key molecule in Wnt/β-Catenin signaling pathway, was targeted by miR-34a-5p in our study, indicating miR-34a-5p could inhibit Wnt/β-Catenin signaling pathway. There is a cross-talk between the regulators of the Wnt/β-catenin and Hippo signaling pathways 34. Hippo signaling pathway has also been reported to involved in tumor progression 35. And the most important, our previous RNA-Seq data showed LEF1 may regulate Hippo pathway 11. The YAP and TAZ proteins function as effectors of the Hippo signaling cascade, essential for tissue development, cell growth, and cell reprogramming 36. Thus, we detected the YAP1/TAZ expression by upregulation of miR-34a-5p in Eca109 and TE1 cells, trying to explore the effect of miR-34a-5p on Hippo pathway. The results showed that YAP1/TAZ expression was downregulated compared to the control groups, indicating that miR-34a-5p inhibited Hippo-YAP1/TAZ signaling pathway in ESCC.

In summary, we determined miR-34a-5p as a tumor suppressor that can block the expression of LEF1, thereby inhibiting proliferation, migration, invasion, and EMT in ESCC. Moreover, miR-34a-5p could inactivate Hippo-YAP1/TAZ signaling pathway to aggravate EMT. Thus, miR-34a-5p and LEF1 may serve as therapeutic targets for ESCC patients.

## Supplementary Material

Supplementary figures.

## Figures and Tables

**Table 1:** The primer sequences of all genes and miRNAs

**LEF1**	
Forward	5′-AACATGGTGGAAAACGAAGC-3′
reverse	5′-GGGTTGGCAGTGATTGTCTT-3′
**E-cadherin**	
forward	5′-CGAGAGCTACACGTTCACGG-3′
reverse	5′-GGGTGTCGAGGGAAAAATAGG-3′
**N-cadherin**	
forward	5′-TTTGATGGAGGTCTCCTAACACC-3′
reverse	5′-ACGTTTAACACGTTGGAAATGTG-3′
**GAPDH**	
forward	5′-TCAAGAAGGTGGTGAAGCAG-3′
reverse	5′-GAGGGGAGATTCAGTGTGGT-3′
**miR-34a-5p mimic**	
forward	5′-TGGCAGTGTCTTAGCTGGTTGT-3′
reverse	5′-GCGAGCACAGAATTAATACGAC-3′
**miR-34a-5p inhibitor**	5'-ACAACCAGCTAAGACACUTCCA -3'
**U6**	
forward	5′-CTCGCTTCGGCAGCACA-3′
reverse	5′-AACGCTTCACGAATTTGCGT-3′

**Table 2:** Correlation between clinicopathological features and expression of miR-34a-5p and LEF1 in patients with ESCC

Characteristics	n	miR-34a-5p relative expression (mean±SD)	P-value	LEF1 mRNA relative expression (mean±SD)	P-value
**Clinicopathological features**			<0.01**		<0.01**
Adjacent tissue	16	1		1	
ESCC tissue	16	0.75 ± 0.30		2.07 ± 0.76	
**Age (years)**			0.47		0.85
≥60	11	0.71 ± 0.10		2.10 ± 0.27	
<60	5	0.83 ± 0.09		2.01 ± 0.14	
**Gender**			0.62		0.99
Female	4	0.82 ± 0.12		2.08 ± 0.16	
Male	12	0.73 ± 0.09		2.07 ± 0.25	
**Pathological T stage**			0.01*		<0.01**
T1+T2	9	0.91 ± 0.09		1.58 ± 0.12	
T3+T4	7	0.55 ± 0.07		2.70 ± 0.26	
**Pathological N stage**			0.03*		0.02*
N0+N1	6	0.95± 0.13		1.54 ± 0.18	
N2+N3	10	0.63 ± 0.07		2.39 ± 0.23	
**Differentiation**			<0.01**		0.03*
G1	7	1.01 ± 0.08		1.62 ± 0.19	
G2+G3	9	0.55 ± 0.06		2.42 ± 0.25	

*P < 0.05, **P < 0.01.

## Abbreviations

ESCC: esophageal squamous cell carcinoma
LEF1: Lymphoid enhancer-binding factor 1
qRT-PCR: Quantitative Real-Time Polymerase Chain Reaction
EMT: epithelial-mesenchymal transition
HEEC: human esophageal epithelium cell
