# Supplementary material for: MiR-34a-5p Inhibits Proliferation, Migration, Invasion and Epithelial-mesenchymal Transition in Esophageal Squamous Cell Carcinoma by Targeting LEF1 and Inactivation of the Hippo-YAP1/TAZ Signaling Pathway
Source: J Cancer. 2020 Mar 4;11(10):3072–81. doi: 10.7150/jca.39861 (PMC7086260; doi:10.7150/jca.39861)

Supplement Fig. S1

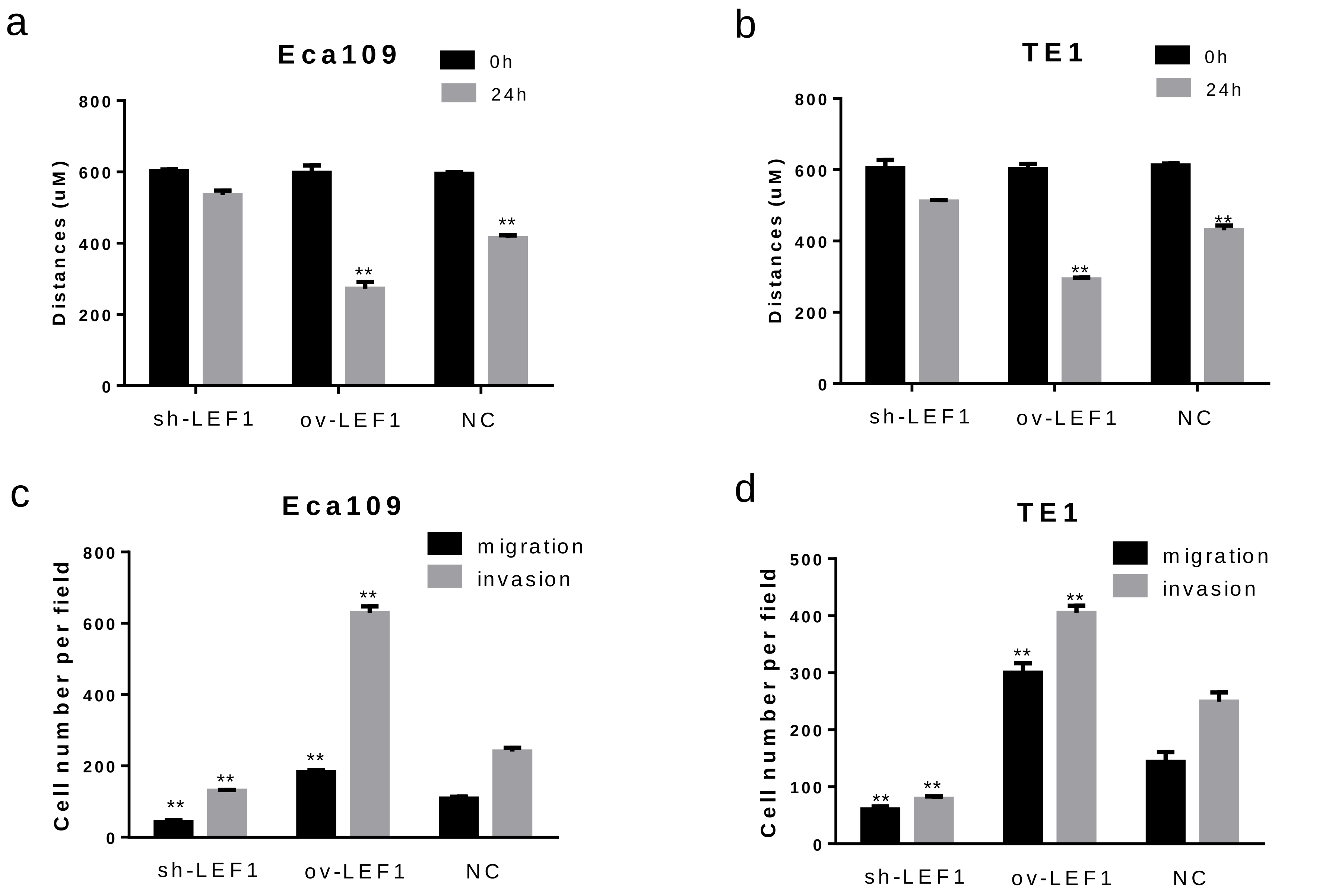

Supplement Fig. S2

Gene Name	MicroRNA	StemLoop ID	miRanda	miRDB	miRWalk	PICTAR4	PICTAR5	Targetscan	SUM
LEF1	hsa-miR-302b	hsa-mir-302b	1	1	1	1	1	1	6
LEF1	hsa-miR-34a	hsa-mir-34a	1	1	1	1	1	1	6
LEF1	hsa-miR-34c-5p	hsa-mir-34c	1	1	1	1	1	1	6
LEF1	hsa-miR-302c	hsa-mir-302c	1	1	1	0	1	1	5
LEF1	hsa-miR-302d	hsa-mir-302d	1	1	1	1	0	1	5
LEF1	hsa-miR-26a	hsa-mir-26a-1	1	0	1	1	1	1	5
LEF1	hsa-miR-302a	hsa-mir-302a	1	0	1	1	1	1	5
LEF1	hsa-miR-26b	hsa-mir-26b	1	0	1	1	1	1	5
LEF1	hsa-miR-372	hsa-mir-372	1	1	1	1	0	1	5
LEF1	hsa-miR-26a	hsa-mir-26a-2	1	0	1	1	1	1	5

Supplement Fig. S3

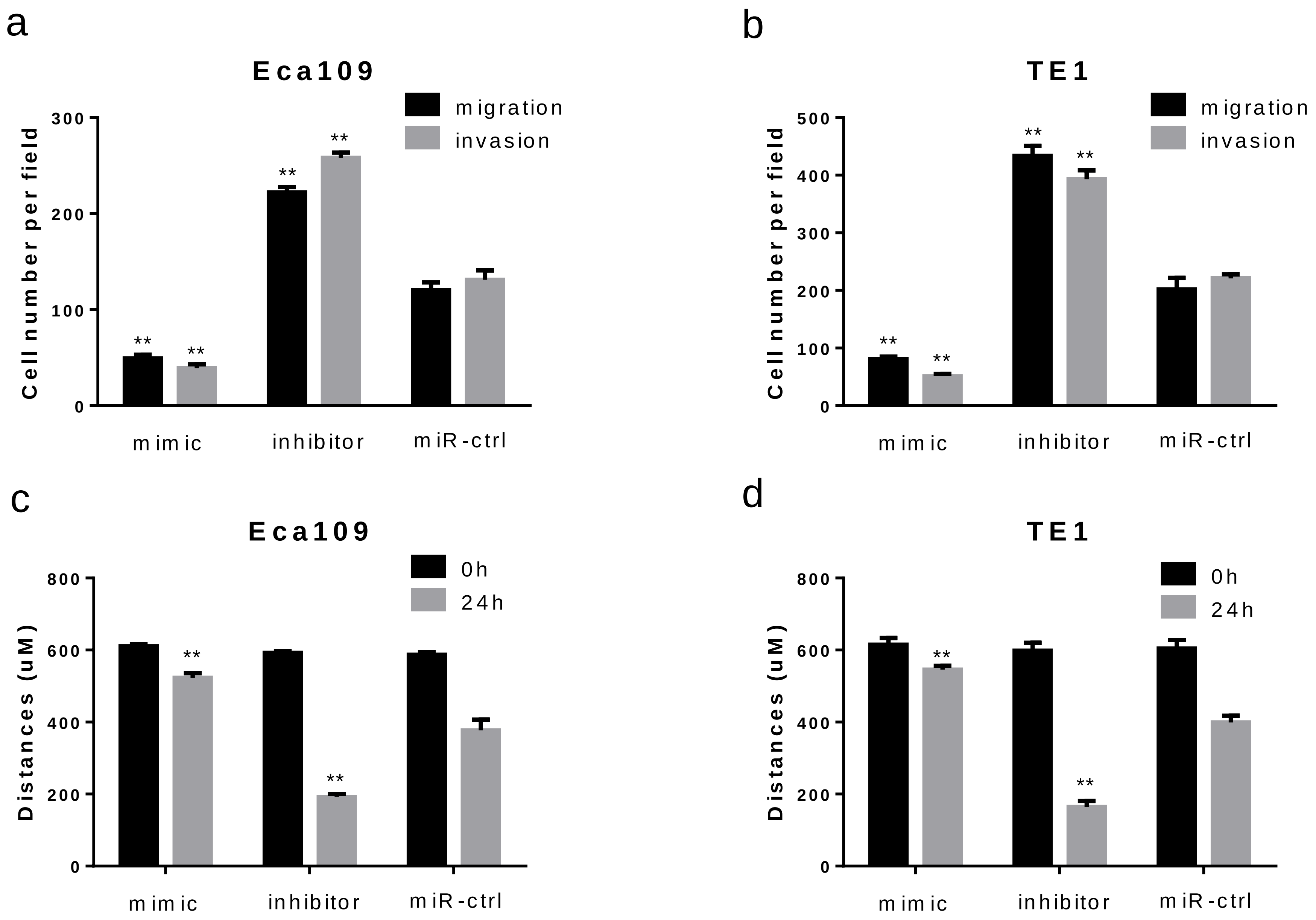

Supplement: Supplementary file 1 — Supplementary figures. [file jcav11p3072s1.pdf]
